# Seasonal Variation in Cases of Acute Appendicitis

**DOI:** 10.1155/2021/8811898

**Published:** 2021-03-02

**Authors:** Rawan A. Rahman AlHarmi, Sayed Ali Almahari, Jasim AlAradi, Asma Alqaseer, Noof Sami AlJirdabi, Fatema Ali Ahmed

**Affiliations:** ^1^Department of Surgery, Salmaniya Medical Complex, Manama, Bahrain; ^2^Department of Pathology, Salmaniya Medical Complex, Manama, Bahrain; ^3^Medical Intern, Salmaniya Medical Complex, Manama, Bahrain

## Abstract

**Objectives:**

To investigate whether the incidence of acute appendicitis increases in summer and whether complicated cases present more in summer.

**Methods:**

A single-center cross-sectional, retrospective study on 697 cases of appendicitis admitted in the year 2018. Inclusion criteria: patients admitted with acute appendicitis who underwent appendectomy of all ages. Exclusion criteria: conservative management. Analysis was performed using Microsoft Excel. Pearson correlation coefficient was calculated to assess the correlation between monthly incidence of appendicitis and mean temperature in that month.

**Results:**

Fifty-one patients who were managed conservatively were excluded. Accordingly, 646 patients were included. Ages ranged from three to 77 years. Males comprised the majority (500, 77.4%). Gangrenous, perforated, and purulent appendices were regarded as complicated appendicitis. The highest number of cases were admitted in summer (234), comprising 36.2% of cases. Complicated cases were equal to 65, of which 23 (35.4%) were admitted in summer and 30 (46.2%) in winter. The highest number of cases was during the month of July (68), while the lowest (40) was during February. This corresponded to the highest recorded mean temperature (36.2°C) and second lowest (19.8°C), respectively. Moderate positive correlation (Pearson's R 0.5183) between the monthly incidence of appendicitis and the mean temperature is noted.

**Conclusion:**

More cases of appendicitis were noted during summer. Monthly incidence correlated positively with the temperature. Larger numbers over several years are needed to draw better conclusions and reach the possible causes behind such variation.

## 1. Introduction

Acute appendicitis is a common global acute abdominal surgical condition [1]. Its etiology is poorly understood. Environmental factors have been implicated, including summer season, air pollution, and dust/allergens. Incidence is increased in certain ethnic groups and complications are increased in minorities, likely due to lack of healthcare access. Cases are classified into simple (nonperforated) and complex (gangrenous, perforated, and pelvic/abdominal abscess) [2–5].

Bahrain is an archipelago in the Gulf with an arid climate. The year is divided into two seasons: summer (June-September) and winter (December-March). These are separated by two transitional periods: first (April, May) and second (October, November) [6].

### 1.1. Purpose

We aim through this study to investigate whether the incidence of acute appendicitis increases in summer season. Our secondary objective is to assess whether the incidence of complicated appendicitis increases in summer season compared to other seasons.

## 2. Methods

### 2.1. Study Design

This is a single-center cross-sectional, retrospective study conducted by the Department of Surgery in Salmaniya Medical Complex (the largest hospital in Bahrain; a 1029-bed tertiary hospital in the capital Manama, serving all citizens and residents of all ages) on 697 cases admitted to our facility with a clinical diagnosis of acute appendicitis in the year 2018. Approval of the institutional ethical review board was attained on 06/07/2020 (no number assigned).

### 2.2. Participants

The patients were selected, as stated previously, from those admitted with acute appendicitis (based on clinical and/or laboratory/imaging diagnosis) in the year 2018. Inclusion criteria: patients admitted with a diagnosis of acute appendicitis who underwent appendectomy (open or laparoscopic) of all ages (including those admitted under pediatric surgery). Exclusion criteria: conservative management (e.g., appendiceal mass and other clinical diagnosis). Patient data was collected from the National Health Information System (I-SEHA). The study did not involve any patient intervention or contact.

Seasons were divided as previously mentioned into winter (January to March, December), first transitional period, termed as T1 (April and May), summer (June to September), and second transitional period, termed as T2 (October and November). Data on the mean monthly temperature was obtained from the official website of the Ministry of Transportation and Telecommunications in Bahrain (Civil Aviation Affairs) [7].

### 2.3. Statistical Analysis

Biostatistical analysis was performed using Microsoft® Excel software for Mac with Analysis ToolPak (Version 16.38). A Chi-Square *P* value of <0.05 was considered statistically significant with a corresponding confidence level of 95%. Pearson correlation coefficient was calculated to assess the correlation between the monthly incidence of acute appendicitis and corresponding mean temperature recorded in each month of the year 2018.

## 3. Results

Out of 697 patients, 51 were managed conservatively and thus excluded. A final total of 646 patients were included in the study. Patients were evaluated by history taking, physical assessment, laboratory investigations, and radiological studies (as indicated). Ages ranged from three to 77 years. Among these, males comprised the majority: 500 (77.4%) compared to 146 (22.6%) females. About 47.4% of the patients originated from Southern Asia countries (India, Bangladesh, Pakistan, Nepal, Sri Lanka), while 40.9% were Bahrainis. Other ethnic groups and demographic data are demonstrated in Table 1.

Final histopathological examination categories are demonstrated in Table 2. Gangrenous, perforated, and purulent appendices were regarded as complicated appendicitis.

The highest number of cases were registered in summer, 234 cases, comprising 36.2% of all cases, while 203 cases were admitted in winter months (31.4%), 106 (16.4%) in T1, and 103 (16%) in T2. Complicated cases were equal to 65, of which 23 (35.4%) were admitted in summer, 30 (46.2%) in winter, seven (10.8%) in T1, and five (7.7%) in T2 (Table 3).

**Figure 1:**
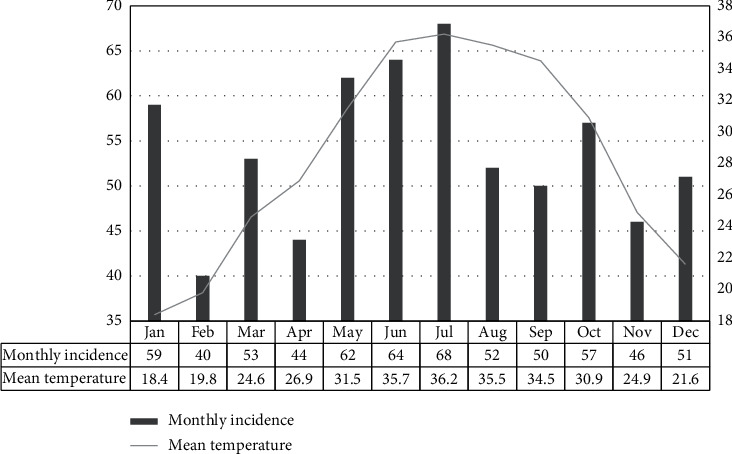
Correlation between monthly incidence and mean recorded temperature.

The highest number of cases was during the month of July (68), while the month with the lowest number was February (40). This corresponded to the highest recorded mean temperature (36.2°C) and the second lowest (19.8°C), respectively. Pearson's correlation coefficient was calculated and revealed a moderate positive correlation (Pearson's R 0.5183; *R*^2^ Coefficient of Determination 0.2686; *P* value 0.084292) between the monthly incidence of appendicitis and the corresponding recorded mean temperature in that month (Figure 1).

We further investigated the variables related to complicated appendicitis, namely, the ethnic group. The highest groups presenting with complications were Bahrainis (23, 35.4%) and Southern Asians (27, 41.5%) (Table 4). Another studied variable was the duration of symptoms at the time of presentation. Two patients were excluded from T1 group, five from the summer group, and two from T2 group due to lack of documentation. For the rest, it was noted that the average time at presentation was 2.03, 2.2, 2.06, and 4.3 days following the onset of symptoms in winter, T1, summer, and T2 groups, respectively (Table 5).

## 4. Discussion

In concordance with many studies, the majority of our patient population were males, accounting for 77.4% of the study population [1].

In our study, the highest number of cases were admitted in summer (234) compared to other seasons. The month with the highest number of cases (68) was July, coinciding with the year's highest mean temperature of 36.2°C recorded in this month. Moreover, our data suggested a moderate positive correlation between the mean temperature and incidence. Our findings are in line with other studies which addressed this issue in Canada, the United States, South Korea, Iran, Italy, Taiwan, Pakistan, Finland, China, and the United Kingdom [3,5,8–13].

On the other hand, a study conducted in northern Saudi Arabia revealed an increased incidence in spring months, which coincide with the sandstorm season. This might be attributed to dust and allergen burden to the lymphoid tissue [4]. In other areas like Nigeria, Turkey, certain parts of the United States and the United Kingdom, different results were obtained. For example, in Nigeria, appendicitis was more common in the rainy season, which is known for humidity, allergens, higher rates of bacterial, viral, and intestinal parasitic infections [5,14,15].

What is the reason behind the observed increase in incidence of appendicitis in summer in many countries? Kaplan et al. have suggested that the exposure to air pollution was noted to trigger the occurrence of appendicitis, particularly in men during summer season [3]. In a study conducted on pediatric age group in Taiwan, it was noted that the percentage of appendicitis cases with fecoliths in summer was lower than other seasons. This was attributed to lymphoid hyperplasia which may be associated with the outbreak of enterovirus during this time of the year [9]. Zhang et al. proposed that pediatric appendicitis cases were associated with higher temperatures, lower humidity, and less sunshine [12].

Dietary habits in summer might play a role. Studies have suggested a role for dehydration and less bowel movements in summer [11]. A review by Fares has stated that the increased consumption of low fiber diet and sugar in summer, particularly when individuals are likely to be out, can lead to constipation and subsequently to appendicitis. Alcohol consumption is reportedly highest during summertime and has been linked to constipation [5]. Though in our part of the world, alcohol consumption is forbidden and restricted for religious reasons.

Another key factor suggested by Fares are the infectious agents implicated in appendicitis through causing lymphoid hyperplasia and resulting lumen obstruction. Summer peak of infection caused by agents such as *Campylobacter*, *Salmonella*, *Escherichia coli*, *Entamoeba histolytica*, *Ascaris lumbricoides*, *Trichuris trichiura*, *Taenia saginata*, *Enterobius vermicularis*, and *Strongyloides stercoralis* is noted in some countries [5]. In our study, only two cases were found on histopathological analysis to have helminths, presented and operated in the months of January and May. Both were found to have nematodes.

We note that 47.4% of our patient population originates from South Asia. They tend to work as labors in industries and might be exposed to air pollution and spend many working hours outdoor in the sun. We believe this might play a role in the disease.

Through our study, we also aimed to investigate whether complicated cases presented more in summer months. However, out of 65 complicated cases, we found that only 23 cases (35.4%) were admitted in summer, 30 (46.2%) in winter, seven (10.8%) in T1, and five (7.7%) in T2.

## 5. Conclusion

In line with other studies, more cases of appendicitis were noted during summer months. The monthly incidence correlated positively with the mean temperature. Larger numbers over several years might be needed to be studied to draw better conclusions on our patient population and even reach to possible causes behind such variation.

## Figures and Tables

**Table 1:** Demographic data of participants.

*Ethnic group*	Number	Percentage (%)
Bahraini	264	40.9
Southern Asians	306	47.4
Other Asians	30	4.6
Gulf nationals	20	3
Other Arabs	9	1.4
African	12	1.9
Hispanic/latino	1	0.15
European	1	0.15
Others	3	0.5
*Sex*
Male	500	77.4
Female	146	22.6
Age	Minimum	Maximum
	3 years	77 years

**Table 2:** Histopathologic classification.

Histopathology	Frequency	Percentage (%)
Acute appendicitis	464	71.8
Early acute appendicitis	62	9.6
Gangrenous appendix	59	9
Reactive lymphoid hyperplasia	37	5.7
Cicatrized/fibrosed appendix	11	1.7
Perforated appendix	5	0.8
Normal appendix	2	0.3
Inflamed appendix with helminth	2	0.3
Purulent with abscess formation	1	0.2
Inflamed appendix with carcinoid tumor	1	0.2
Granulomatous appendicitis	1	0.2
Inflamed appendix with sessile serrated adenoma (SSA)	1	0.2

**Table 3:** Distribution of cases by season.

Season	Months	All cases (*N* = 646) (%)	Complicated cases (*N* = 65, 10%) (%)	Non-complicated cases (*N* = 581, 90%) (%)
Winter	January	203 (31.4)	30 (46.2)	173 (53.8)
	February			
	March			
	December			
First transitional period (T1)	April	106 (16.4)	7 (10.8)	99 (89.2)
	May			
Summer	June	234 (36.2)	23 (35.4)	211 (64.6)
	July			
	August			
	September			
Second transitional period (T2)	October	103 (16)	5 (7.7)	98 (92.3)
	November			

**Table 4:** Complicated cases by ethnic group.

Ethnic group	Number	Percentage (%)	Percentage from ethnic group overall (%)
Bahraini	23	35.4	8.7
Southern asians	27	41.5	8.8
Other asians	8	12.3	26.7
Gulf nationals	2	3.1	10
Other arabs	2	3.1	22.2
African	2	3.1	16.7
Hispanic/latino	0	0	0
European	1	1.5	100
Others	0	0	0

**Table 5:** Duration of symptoms (in days) at presentation by season for complicated cases.

Season	Average	Maximum	Minimum
Winter	2.03	7	1
T1	2.2	4	1
Summer	2.06	4	1
T2	4.3	10	1

## Data Availability

The data are available in the supplementary information files.
