# Supplementary material for: Seasonal Variation in Cases of Acute Appendicitis
Source: Surg Res Pract. 2021 Mar 2;2021:8811898. doi: 10.1155/2021/8811898 (PMC7943315; doi:10.1155/2021/8811898)
Supplement: Supplementary Materials — Raw data are provided. [file 8811898.f1.pdf]

Season	Sex	Age	Ethnicity	Date of Admission	Histopathology	Duration in days (Complicated)
W	M	17	BAH	1-Jan-18	Gangrenous appendix	1
	M	15	BAH	1-Jan-18	Normal	
	M	39	Bangladeshi	2-Jan-18	Acute appendicitis	
	M	19	BAH	2-Jan-18	Gangrenous appendix	1
	M	40	Pakistani	3-Jan-18	Acute appendicitis	
	M	29	Pakistani	3-Jan-18	Acute appendicitis	
	M	29	Bangladeshi	3-Jan-18	Acute appendicitis	
	M	25	Indian	3-Jan-18	Gangrenous appendix	1
	M	22	BAH	3-Jan-18	Acute appendicitis	
	M	12	Bangladeshi	3-Jan-18	Acute appendicitis	
	M	22	Indian	4-Jan-18	Acute appendicitis	
	F	38	BAH	5-Jan-18	Acute appendicitis	
	F	38	Philipino	5-Jan-18	Acute appendicitis	
	F	45	Philipino	6-Jan-18	Acute appendicitis	
	M	19	Indian	6-Jan-18	Early acute appendicitis	
	M	31	Bangladeshi	6-Jan-18	Acute appendicitis	
	M	28	Pakistani	7-Jan-18	Acute appendicitis	
	M	26	GCC	7-Jan-18	Acute appendicitis	
	F	7	BAH	7-Jan-18	Acute appendicitis	
	F	33	GCC	8-Jan-18	Acute appendicitis	
	M	28	BAH	8-Jan-18	Early acute appendicitis	
	M	22	Pakistani	9-Jan-18	Gangrenous appendix	2
	M	37	Moroccan	11-Jan-18	Early acute appendicitis	
	F	28	African	11-Jan-18	Reactive lymphoid hyperplasia	
	M	31	Indian	11-Jan-18	Acute appendicitis	
	F	26	Colombian	11-Jan-18	Acute appendicitis	
	M	21	Indian	13-Jan-18	Acute appendicitis	
	M	12	Egyptian	13-Jan-18	Acute appendicitis	
	F	31	BAH	14-Jan-18	Acute appendicitis	
	M	20	BAH	14-Jan-18	Acute appendicitis	
	M	11	BAH	14-Jan-18	Helminth	
	M	32	Indian	15-Jan-18	Acute appendicitis	
	M	24	BAH	16-Jan-18	Gangrenous appendix	1
	M	18	Indian	16-Jan-18	Acute appendicitis	
	F	14	BAH	17-Jan-18	Early acute appendicitis	
	F	19	Yemeni	19-Jan-18	Early acute appendicitis	
	F	14	BAH	20-Jan-18	Gangrenous appendix	1
	M	16	BAH	21-Jan-18	Acute appendicitis	
	F	18	BAH	21-Jan-18	Acute appendicitis	
	M	29	Indian	21-Jan-18	Acute appendicitis	
	M	10	BAH	22-Jan-18	Acute appendicitis	
	M	31	Indian	23-Jan-18	Gangrenous appendix	1
	M	29	Philipino	23-Jan-18	Gangrenous appendix	3
	M	28	Bangladeshi	23-Jan-18	Acute appendicitis	
	M	18	BAH	24-Jan-18	Acute appendicitis	
	M	38	Bangladeshi	25-Jan-18	Cicatrized	
	F	35	Thai	25-Jan-18	Acute appendicitis	
	F	37	Indonesian	26-Jan-18	Gangrenous appendix	7
	M	33	Bangladeshi	27-Jan-18	Acute appendicitis	

M	13	BAH	27-Jan-18	Acute appendicitis	
M	22	BAH	28-Jan-18	Acute appendicitis	
M	77	Iranian	28-Jan-18	Perforated appendix	3
F	22	BAH	29-Jan-18	Acute appendicitis	
M	47	Pakistani	29-Jan-18	Acute appendicitis	
F	33	Thai	29-Jan-18	Perforated appendix	3
M	29	Bangladeshi	30-Jan-18	Gangrenous appendix	3
M	39	BAH	30-Jan-18	Acute appendicitis	
M	30	Indian	30-Jan-18	Acute appendicitis	
M	29	Bangladeshi	31-Jan-18	Early acute appendicitis	
M	31	Bangladeshi	1-Feb-18	Gangrenous appendix	1
M	24	Indian	2-Feb-18	Acute appendicitis	
M	23	Indian	4-Feb-18	Acute appendicitis	
M	35	Bangladeshi	5-Feb-18	Acute appendicitis	
M	43	African	5-Feb-18	Gangrenous appendix	1
M	23	Indian	6-Feb-18	Gangrenous appendix	1
M	13	BAH	7-Feb-18	Acute appendicitis	
M	43	Bangladeshi	9-Feb-18	Reactive lymphoid hyperplasia	
M	17	BAH	9-Feb-18	Acute appendicitis	
M	11	BAH	10-Feb-18	Acute appendicitis	
M	33	Bangladeshi	11-Feb-18	Acute appendicitis	
M	35	Bangladeshi	11-Feb-18	Acute appendicitis	
M	6	BAH	11-Feb-18	Early acute appendicitis	
M	23	GCC	12-Feb-18	Acute appendicitis	
M	35	Indian	12-Feb-18	Acute appendicitis	
M	30	Bangladeshi	12-Feb-18	Acute appendicitis	
F	7	BAH	12-Feb-18	Reactive lymphoid hyperplasia	
M	32	Indian	13-Feb-18	Acute appendicitis	
F	32	Indian	14-Feb-18	Acute appendicitis	
M	28	BAH	15-Feb-18	Acute appendicitis	
M	33	Indian	15-Feb-18	Acute appendicitis	
M	23	Pakistani	16-Feb-18	Acute appendicitis	
M	32	Bangladeshi	16-Feb-18	Acute appendicitis	
M	63	Indian	17-Feb-18	Acute appendicitis	
F	11	BAH	17-Feb-18	Acute appendicitis	
M	33	Bangladeshi	19-Feb-18	Early acute appendicitis	
M	37	Bangladeshi	19-Feb-18	Acute appendicitis	
M	36	African	19-Feb-18	Reactive lymphoid hyperplasia	
M	29	Bangladeshi	20-Feb-18	Early acute appendicitis	
M	52	BAH	23-Feb-18	Acute appendicitis	
M	29	Indian	23-Feb-18	Acute appendicitis	
F	15	BAH	23-Feb-18	Acute appendicitis	
F	32	Pakistani	23-Feb-18	Acute appendicitis	
M	9	BAH	23-Feb-18	Acute appendicitis	
F	28	BAH	24-Feb-18	Acute appendicitis	
M	24	BAH	26-Feb-18	Acute appendicitis	
M	43	BAH	26-Feb-18	Acute appendicitis	
M	16	BAH	27-Feb-18	Acute appendicitis	
F	27	Philipino	28-Feb-18	Acute appendicitis	
M	45	Pakistani	28-Feb-18	Gangrenous appendix	2
M	29	Bangladeshi	1-Mar-18	Reactive lymphoid hyperplasia	
M	31	Bangladeshi	1-Mar-18	Gangrenous appendix	3

	M	29	Bangladeshi	1-Mar-18	Early acute appendicitis	
	M	13	Indian	1-Mar-18	Reactive lymphoid hyperplasia	
	M	27	Bangladeshi	2-Mar-18	Acute appendicitis	
	F	3	BAH	3-Mar-18	Acute appendicitis	
	M	22	Pakistani	4-Mar-18	Acute appendicitis	
	F	52	BAH	5-Mar-18	Acute appendicitis	
	M	31	BAH	6-Mar-18	Acute appendicitis	
	M	29	GCC	6-Mar-18	Acute appendicitis	
	M	19	Indian	7-Mar-18	Gangrenous appendix	2
	M	55	BAH	7-Mar-18	Acute appendicitis	
	F	24	BAH	8-Mar-18	Early acute appendicitis	
	M	34	Bangladeshi	8-Mar-18	Perforated appendix	1
	M	6	BAH	8-Mar-18	Acute appendicitis	
	M	39	Pakistani	9-Mar-18	Perforated appendix	1
	M	28	Bangladeshi	11-Mar-18	Acute appendicitis	
	F	16	BAH	12-Mar-18	Acute appendicitis	
	M	38	Pakistani	14-Mar-18	Gangrenous appendix	1
	F	30	African	14-Mar-18	Reactive lymphoid hyperplasia	
	F	35	GCC	14-Mar-18	Acute appendicitis	
	M	48	BAH	14-Mar-18	Acute appendicitis	
	M	27	BAH	15-Mar-18	Acute appendicitis	
	M	30	Pakistani	17-Mar-18	Acute appendicitis	
	M	35	BAH	17-Mar-18	Acute appendicitis	
	M	11	Indian	18-Mar-18	Acute appendicitis	
	M	40	Pakistani	19-Mar-18	Acute appendicitis	
	M	19	BAH	19-Mar-18	Acute appendicitis	
	M	12	BAH	19-Mar-18	Gangrenous appendix	4
	M	25	BAH	20-Mar-18	Early acute appendicitis	
	M	35	Philipino	21-Mar-18	Acute appendicitis	
	M	38	BAH	21-Mar-18	Acute appendicitis	
	M	17	GCC	21-Mar-18	Acute appendicitis	
	M	16	BAH	21-Mar-18	Acute appendicitis	
	F	12	BAH	21-Mar-18	Early acute appendicitis	
	M	3	BAH	21-Mar-18	Gangrenous appendix	6
	M	44	Bangladeshi	22-Mar-18	Acute appendicitis	
	F	31	BAH	22-Mar-18	Acute appendicitis	
	F	30	Philipino	22-Mar-18	Early acute appendicitis	
	M	28	Bangladeshi	22-Mar-18	Acute appendicitis	
	M	27	Philipino	23-Mar-18	Acute appendicitis	
	F	25	BAH	23-Mar-18	Early acute appendicitis	
	F	23	BAH	23-Mar-18	Early acute appendicitis	
	F	44	Philipino	23-Mar-18	Early acute appendicitis	
	F	16	BAH	23-Mar-18	Early acute appendicitis	
	M	13	BAH	23-Mar-18	Gangrenous appendix	1
	M	21	BAH	24-Mar-18	Acute appendicitis	
	M	37	Bangladeshi	25-Mar-18	Acute appendicitis	
	M	5	BAH	25-Mar-18	Acute appendicitis	
	F	30	Thai	28-Mar-18	Early acute appendicitis	
	F	24	BAH	29-Mar-18	Acute appendicitis	
	M	31	BAH	29-Mar-18	Acute appendicitis	
	F	27	BAH	31-Mar-18	Acute appendicitis	
T1	M	26	Bangladeshi	2-Apr-18	Acute appendicitis	

M	51	BAH	4-Apr-18	Acute appendicitis	
M	11	BAH	4-Apr-18	Reactive lymphoid hyperplasia	
M	35	Bangladeshi	5-Apr-18	Acute appendicitis	
F	10	Bangladeshi	5-Apr-18	Acute appendicitis	
F	32	Indian	6-Apr-18	Early acute appendicitis	
M	41	Indian	7-Apr-18	Acute appendicitis	
M	9	BAH	7-Apr-18	Acute appendicitis	
M	33	Bangladeshi	9-Apr-18	Acute appendicitis	
M	31	Pakistani	9-Apr-18	Acute appendicitis	
M	48	Bangladeshi	9-Apr-18	Acute appendicitis	
M	34	Bangladeshi	10-Apr-18	Acute appendicitis	
M	6	BAH	10-Apr-18	Gangrenous appendix	3
M	24	Pakistani	11-Apr-18	Acute appendicitis	
M	43	BAH	11-Apr-18	Acute appendicitis	
M	34	Bangladeshi	12-Apr-18	Acute appendicitis	
M	15	BAH	12-Apr-18	Acute appendicitis	
F	10	Bangladeshi	13-Apr-18	Acute appendicitis	
M	28	Bangladeshi	14-Apr-18	Acute appendicitis	
M	44	Pakistani	14-Apr-18	Acute appendicitis	
F	16	Indian	15-Apr-18	Acute appendicitis	
M	38	Bangladeshi	15-Apr-18	Acute appendicitis	
F	42	BAH	17-Apr-18	Cicatrized	
M	14	BAH	17-Apr-18	Acute appendicitis	
M	36	Indian	17-Apr-18	Reactive lymphoid hyperplasia	
M	41	Indian	18-Apr-18	Acute appendicitis	
M	29	Indian	19-Apr-18	Acute appendicitis	
M	18	BAH	19-Apr-18	Acute appendicitis	
M	31	Bangladeshi	21-Apr-18	Early acute appendicitis	
F	55	BAH	22-Apr-18	Acute appendicitis	
F	14	BAH	22-Apr-18	Reactive lymphoid hyperplasia	
F	26	BAH	22-Apr-18	Early acute appendicitis	
M	35	Bangladeshi	23-Apr-18	Acute appendicitis	
M	37	Indian	24-Apr-18	Acute appendicitis	
M	28	BAH	24-Apr-18	Acute appendicitis	
M	32	Philipino	24-Apr-18	Gangrenous appendix	1
M	19	BAH	25-Apr-18	Acute appendicitis	
M	23	Pakistani	25-Apr-18	Acute appendicitis	
F	33	BAH	25-Apr-18	Acute appendicitis	
M	28	Indian	26-Apr-18	Acute appendicitis	
M	30	Philipino	28-Apr-18	Acute appendicitis	
F	12	BAH	28-Apr-18	Early acute appendicitis	
M	29	Indian	30-Apr-18	Acute appendicitis	
M	22	Nepalese	30-Apr-18	Early acute appendicitis	
M	30	Indian	2-May-18	Early acute appendicitis	
M	40	Indian	2-May-18	Early acute appendicitis	
F	8	BAH	2-May-18	Acute appendicitis	
M	13	GCC	3-May-18	Acute appendicitis	
M	50	Bangladeshi	4-May-18	Reactive lymphoid hyperplasia	
M	35	Bangladeshi	4-May-18	Acute appendicitis	
M	22	BAH	4-May-18	Acute appendicitis	
M	26	BAH	4-May-18	Acute appendicitis	
M	17	BAH	4-May-18	Acute appendicitis	

M	17	BAH	5-May-18	Acute appendicitis	
M	20	BAH	5-May-18	Gangrenous appendix	Not specified
F	18	BAH	6-May-18	Helminth	
M	25	BAH	6-May-18	Acute appendicitis	
M	10	BAH	6-May-18	Reactive lymphoid hyperplasia	
M	30	Nepalese	7-May-18	Acute appendicitis	
F	16	Other	7-May-18	Reactive lymphoid hyperplasia	
M	31	Pakistani	8-May-18	Acute appendicitis	
F	27	African	8-May-18	Gangrenous appendix	2
M	28	Indian	8-May-18	Early acute appendicitis	
M	39	Bangladeshi	8-May-18	Acute appendicitis	
M	29	Bangladeshi	9-May-18	Acute appendicitis	
M	36	Bangladeshi	9-May-18	Acute appendicitis	
M	14	BAH	9-May-18	Acute appendicitis	
M	9	BAH	9-May-18	Acute appendicitis	
M	55	BAH	10-May-18	Acute appendicitis	
M	7	BAH	12-May-18	Acute appendicitis	
M	35	BAH	13-May-18	Acute appendicitis	
F	21	BAH	13-May-18	Acute appendicitis	
M	31	Indian	14-May-18	Acute appendicitis	
M	27	Bangladeshi	14-May-18	Acute appendicitis	
M	33	GCC	15-May-18	Acute appendicitis	
M	23	Pakistani	16-May-18	Acute appendicitis	
M	37	Indian	17-May-18	Acute appendicitis	
M	40	Bangladeshi	17-May-18	Cicatrized	
M	58	Indian	18-May-18	Acute appendicitis	
M	20	Indian	20-May-18	Acute appendicitis	
M	14	Pakistani	20-May-18	Acute appendicitis	
M	62	Indian	21-May-18	Acute appendicitis	
M	29	BAH	21-May-18	Acute appendicitis	
M	27	Indian	21-May-18	Acute appendicitis	
M	36	Pakistani	22-May-18	Acute appendicitis	
M	19	BAH	22-May-18	Acute appendicitis	
M	56	Indian	22-May-18	Acute appendicitis	
M	36	BAH	23-May-18	Early acute appendicitis	
M	43	Indian	23-May-18	Acute appendicitis	
M	39	BAH	23-May-18	Acute appendicitis	
M	25	Pakistani	25-May-18	Acute appendicitis	
M	14	GCC	25-May-18	Reactive lymphoid hyperplasia	
M	36	Bangladeshi	25-May-18	Acute appendicitis	
F	38	BAH	25-May-18	Gangrenous appendix	Not specified
M	8	BAH	25-May-18	Acute appendicitis	
F	19	BAH	26-May-18	Early acute appendicitis	
M	39	BAH	26-May-18	Acute appendicitis	
F	26	BAH	26-May-18	Gangrenous appendix	1
M	41	Bangladeshi	27-May-18	Acute appendicitis	
M	12	BAH	27-May-18	Acute appendicitis	
F	12	BAH	28-May-18	Reactive lymphoid hyperplasia	
M	22	BAH	29-May-18	Acute appendicitis	
M	34	BAH	30-May-18	Acute appendicitis	
M	27	Pakistani	30-May-18	Acute appendicitis	
M	5	BAH	30-May-18	Acute appendicitis	

	M	32	BAH	31-May-18	Perforated appendix	4
S	F	19	Philipino	3-Jun-18	Acute appendicitis	
	M	21	Indian	3-Jun-18	Early acute appendicitis	
	M	35	Bangladeshi	4-Jun-18	Acute appendicitis	
	M	28	Indian	4-Jun-18	Acute appendicitis	
	M	29	BAH	6-Jun-18	Acute appendicitis	
	M	22	BAH	6-Jun-18	Acute appendicitis	
	M	32	Bangladeshi	6-Jun-18	Acute appendicitis	
	F	33	Philipino	6-Jun-18	Acute appendicitis	
	M	30	Pakistani	8-Jun-18	Acute appendicitis	
	M	34	Bangladeshi	9-Jun-18	Gangrenous appendix	2
	M	22	BAH	9-Jun-18	Acute appendicitis	
	M	51	BAH	9-Jun-18	Acute appendicitis	
	M	28	Bangladeshi	9-Jun-18	Acute appendicitis	
	M	10	BAH	9-Jun-18	Acute appendicitis	
	M	31	Bangladeshi	10-Jun-18	Acute appendicitis	
	M	14	BAH	10-Jun-18	Cicatrized	
	M	50	BAH	10-Jun-18	Gangrenous appendix	3
	F	31	BAH	11-Jun-18	Early acute appendicitis	
	M	37	Pakistani	11-Jun-18	Acute appendicitis	
	F	9	BAH	11-Jun-18	Acute appendicitis	
	M	36	Pakistani	12-Jun-18	Acute appendicitis	
	M	31	Bangladeshi	12-Jun-18	Acute appendicitis	
	M	64	BAH	12-Jun-18	Acute appendicitis	
	M	28	Nepalese	12-Jun-18	Gangrenous appendix	1
	F	20	BAH	13-Jun-18	Reactive lymphoid hyperplasia	
	M	32	BAH	13-Jun-18	Reactive lymphoid hyperplasia	
	M	30	Pakistani	14-Jun-18	Acute appendicitis	
	M	36	Indian	15-Jun-18	Acute appendicitis	
	M	23	BAH	15-Jun-18	Acute appendicitis	
	M	24	Pakistani	16-Jun-18	Early acute appendicitis	
	M	26	Pakistani	16-Jun-18	Acute appendicitis	
	M	31	Pakistani	17-Jun-18	Acute appendicitis	
	M	28	Bangladeshi	17-Jun-18	Acute appendicitis	
	F	5	BAH	17-Jun-18	Granulomatous appendicitis	
	M	30	GCC	18-Jun-18	Gangrenous appendix	1
	M	37	Other	18-Jun-18	Acute appendicitis	
	M	29	Indian	18-Jun-18	Acute appendicitis	
	M	36	Indian	19-Jun-18	Gangrenous appendix	Not specified
	M	29	Bangladeshi	19-Jun-18	Acute appendicitis	
	M	27	BAH	20-Jun-18	Gangrenous appendix	Not specified
	F	14	BAH	20-Jun-18	Acute appendicitis	
	F	7	BAH	20-Jun-18	Acute appendicitis	
	M	28	Pakistani	21-Jun-18	Acute appendicitis	
	M	24	Indian	21-Jun-18	Acute appendicitis	
	F	31	Pakistani	21-Jun-18	Acute appendicitis	
	F	36	GCC	21-Jun-18	Acute appendicitis	
	M	59	Indian	21-Jun-18	Abscess	
	M	13	BAH	22-Jun-18	Acute appendicitis	
	F	15	BAH	23-Jun-18	Acute appendicitis	
	M	50	Indian	23-Jun-18	Acute appendicitis	
	M	36	BAH	23-Jun-18	Acute appendicitis	

M	58	BAH	24-Jun-18	Acute appendicitis	
M	34	BAH	24-Jun-18	Gangrenous appendix	2
M	27	Indian	24-Jun-18	Acute appendicitis	
M	27	BAH	25-Jun-18	Acute appendicitis	
M	27	Indian	25-Jun-18	Acute appendicitis	
M	23	Indian	25-Jun-18	Acute appendicitis	
M	29	Indian	25-Jun-18	Acute appendicitis	
M	11	BAH	25-Jun-18	Gangrenous appendix	4
F	25	GCC	26-Jun-18	Reactive lymphoid hyperplasia	
M	19	BAH	26-Jun-18	Acute appendicitis	
M	37	Indian	29-Jun-18	Reactive lymphoid hyperplasia	
M	30	Bangladeshi	30-Jun-18	Acute appendicitis	
M	27	Bangladeshi	30-Jun-18	Acute appendicitis	
M	28	Indian	1-Jul-18	Acute appendicitis	
M	28	BAH	1-Jul-18	Acute appendicitis	
M	23	Pakistani	1-Jul-18	Reactive lymphoid hyperplasia	
F	40	BAH	2-Jul-18	Acute appendicitis	
F	22	BAH	2-Jul-18	Reactive lymphoid hyperplasia	
M	9	BAH	2-Jul-18	Acute appendicitis	
M	8	BAH	2-Jul-18	Acute appendicitis	
M	28	BAH	3-Jul-18	Acute appendicitis	
M	23	BAH	3-Jul-18	Cicatrized	
M	34	Bangladeshi	3-Jul-18	Acute appendicitis	
M	39	Indian	4-Jul-18	Acute appendicitis	
M	9	BAH	5-Jul-18	Carcinoid	
F	25	BAH	6-Jul-18	Early acute appendicitis	
F	26	BAH	6-Jul-18	Early acute appendicitis	
M	35	Bangladeshi	6-Jul-18	Gangrenous appendix	1
M	35	Bangladeshi	7-Jul-18	Acute appendicitis	
M	14	BAH	7-Jul-18	Early acute appendicitis	
M	45	Philipino	8-Jul-18	Acute appendicitis	
M	36	BAH	10-Jul-18	Acute appendicitis	
M	26	Indian	10-Jul-18	Acute appendicitis	
M	49	Indian	10-Jul-18	Acute appendicitis	
M	30	Bangladeshi	11-Jul-18	Acute appendicitis	
M	24	Indian	11-Jul-18	Gangrenous appendix	2
F	6	Bangladeshi	11-Jul-18	Acute appendicitis	
M	26	Pakistani	12-Jul-18	Acute appendicitis	
M	30	Pakistani	12-Jul-18	Acute appendicitis	
M	29	Bangladeshi	13-Jul-18	Acute appendicitis	
M	25	Pakistani	13-Jul-18	Acute appendicitis	
M	44	BAH	13-Jul-18	Acute appendicitis	
M	34	BAH	14-Jul-18	Acute appendicitis	
M	29	Pakistani	14-Jul-18	Acute appendicitis	
M	35	GCC	14-Jul-18	Acute appendicitis	
M	21	Indian	15-Jul-18	Acute appendicitis	
M	33	Philipino	15-Jul-18	Gangrenous appendix	2
M	39	BAH	17-Jul-18	Early acute appendicitis	
M	27	BAH	17-Jul-18	Acute appendicitis	
M	23	BAH	18-Jul-18	Acute appendicitis	
M	36	Bangladeshi	18-Jul-18	Acute appendicitis	
M	27	Indian	18-Jul-18	Reactive lymphoid hyperplasia	

M	8	BAH	18-Jul-18	Acute appendicitis	
M	22	Indian	19-Jul-18	Acute appendicitis	
M	27	Bangladeshi	19-Jul-18	Acute appendicitis	
F	28	BAH	20-Jul-18	Acute appendicitis	
F	22	BAH	20-Jul-18	Acute appendicitis	
F	34	Bangladeshi	20-Jul-18	Early acute appendicitis	
F	29	BAH	20-Jul-18	Early acute appendicitis	
M	32	Indian	21-Jul-18	Acute appendicitis	
F	31	Indian	22-Jul-18	Acute appendicitis	
F	49	Philipino	23-Jul-18	Gangrenous appendix	3
F	6	BAH	23-Jul-18	Acute appendicitis	
M	29	African	24-Jul-18	Acute appendicitis	
M	31	Bangladeshi	24-Jul-18	Early acute appendicitis	
M	26	BAH	24-Jul-18	Acute appendicitis	
M	30	Bangladeshi	24-Jul-18	Acute appendicitis	
F	18	BAH	24-Jul-18	Acute appendicitis	
M	32	Bangladeshi	25-Jul-18	Reactive lymphoid hyperplasia	
M	35	Bangladeshi	25-Jul-18	Acute appendicitis	
F	20	BAH	25-Jul-18	Reactive lymphoid hyperplasia	
M	28	BAH	25-Jul-18	Acute appendicitis	
M	34	Indian	26-Jul-18	Acute appendicitis	
M	37	Indian	29-Jul-18	Acute appendicitis	
M	28	Indian	29-Jul-18	Acute appendicitis	
M	21	Pakistani	29-Jul-18	Acute appendicitis	
M	28	Indian	30-Jul-18	Acute appendicitis	
F	26	African	31-Jul-18	Acute appendicitis	
M	29	Bangladeshi	31-Jul-18	Acute appendicitis	
M	30	Pakistani	31-Jul-18	Acute appendicitis	
M	35	Bangladeshi	31-Jul-18	Acute appendicitis	
M	32	Bangladeshi	1-Aug-18	Acute appendicitis	
M	39	Bangladeshi	3-Aug-18	Acute appendicitis	
M	37	BAH	3-Aug-18	Acute appendicitis	
M	26	Pakistani	3-Aug-18	Acute appendicitis	
M	36	Nepalese	4-Aug-18	Acute appendicitis	
M	28	Pakistani	4-Aug-18	Gangrenous appendix	1
M	42	BAH	5-Aug-18	Acute appendicitis	
F	38	Egyptian	5-Aug-18	Acute appendicitis	
F	25	Thai	7-Aug-18	Acute appendicitis	
M	26	Pakistani	7-Aug-18	Acute appendicitis	
F	34	Other	7-Aug-18	Acute appendicitis	
M	26	Pakistani	8-Aug-18	Acute appendicitis	
M	7	BAH	8-Aug-18	Acute appendicitis	
M	31	GCC	9-Aug-18	Acute appendicitis	
F	31	GCC	10-Aug-18	Acute appendicitis	
M	23	Indian	10-Aug-18	Early acute appendicitis	
M	28	Bangladeshi	10-Aug-18	Acute appendicitis	
M	27	Pakistani	10-Aug-18	Cicatrized	
M	17	BAH	10-Aug-18	Acute appendicitis	
M	10	GCC	10-Aug-18	Gangrenous appendix	Not specified
M	28	Bangladeshi	11-Aug-18	Acute appendicitis	
M	22	Pakistani	11-Aug-18	Acute appendicitis	
M	28	Bangladeshi	11-Aug-18	Acute appendicitis	

F	29	Burmese	11-Aug-18	Acute appendicitis	
M	36	Indian	12-Aug-18	Acute appendicitis	
F	38	BAH	14-Aug-18	Acute appendicitis	
M	56	Indian	14-Aug-18	Acute appendicitis	
M	19	Pakistani	14-Aug-18	Early acute appendicitis	
M	23	Indian	14-Aug-18	Gangrenous appendix	2
M	24	Pakistani	15-Aug-18	Gangrenous appendix	1
M	35	Bangladeshi	16-Aug-18	Acute appendicitis	
M	19	BAH	17-Aug-18	Acute appendicitis	
M	27	BAH	18-Aug-18	Acute appendicitis	
M	30	Pakistani	21-Aug-18	Acute appendicitis	
M	57	BAH	22-Aug-18	Acute appendicitis	
F	6	BAH	24-Aug-18	Acute appendicitis	
M	34	Bangladeshi	25-Aug-18	Acute appendicitis	
M	10	BAH	25-Aug-18	Reactive lymphoid hyperplasia	
M	26	BAH	26-Aug-18	Acute appendicitis	
M	11	BAH	27-Aug-18	Early acute appendicitis	
M	12	BAH	27-Aug-18	Acute appendicitis	
F	22	BAH	28-Aug-18	Reactive lymphoid hyperplasia	
M	53	Bangladeshi	28-Aug-18	Acute appendicitis	
F	5	BAH	28-Aug-18	Acute appendicitis	
M	34	Srilankan	29-Aug-18	Acute appendicitis	
M	32	Indian	30-Aug-18	Acute appendicitis	
F	48	BAH	30-Aug-18	Early acute appendicitis	
M	9	BAH	30-Aug-18	Acute appendicitis	
F	32	BAH	31-Aug-18	Acute appendicitis	
F	17	BAH	31-Aug-18	Acute appendicitis	
M	36	BAH	31-Aug-18	Acute appendicitis	
M	7	BAH	31-Aug-18	Acute appendicitis	
F	35	Jordanian	2-Sep-18	Gangrenous appendix	2
M	61	Indian	2-Sep-18	Acute appendicitis	
M	40	Bangladeshi	2-Sep-18	Gangrenous appendix	2
F	31	BAH	2-Sep-18	Acute appendicitis	
F	26	Moroccan	3-Sep-18	Cicatrized	
M	12	BAH	3-Sep-18	Acute appendicitis	
M	33	BAH	4-Sep-18	Acute appendicitis	
M	38	Nepalese	4-Sep-18	Acute appendicitis	
M	9	BAH	4-Sep-18	Acute appendicitis	
M	22	Pakistani	5-Sep-18	Acute appendicitis	
F	22	Kazakhstani	5-Sep-18	Reactive lymphoid hyperplasia	
M	15	BAH	5-Sep-18	Reactive lymphoid hyperplasia	
M	32	Bangladeshi	5-Sep-18	Acute appendicitis	
M	28	Nepalese	7-Sep-18	Acute appendicitis	
M	38	GCC	7-Sep-18	Acute appendicitis	
F	9	BAH	7-Sep-18	Gangrenous appendix	2
M	31	BAH	8-Sep-18	Acute appendicitis	
M	25	Indian	9-Sep-18	Acute appendicitis	
F	10	BAH	9-Sep-18	Acute appendicitis	
F	10	Indian	9-Sep-18	Acute appendicitis	
F	31	BAH	10-Sep-18	Acute appendicitis	
M	37	Bangladeshi	11-Sep-18	Acute appendicitis	
F	32	BAH	12-Sep-18	Acute appendicitis	

	M	9	BAH	12-Sep-18	Gangrenous appendix	3
	M	35	Bangladeshi	13-Sep-18	Reactive lymphoid hyperplasia	
	M	32	Pakistani	13-Sep-18	Acute appendicitis	
	M	11	BAH	13-Sep-18	Acute appendicitis	
	F	30	BAH	14-Sep-18	Acute appendicitis	
	M	21	Pakistani	16-Sep-18	Gangrenous appendix	3
	M	23	Pakistani	17-Sep-18	Acute appendicitis	
	F	15	BAH	18-Sep-18	Early acute appendicitis	
	M	21	BAH	18-Sep-18	Acute appendicitis	
	M	23	Indian	18-Sep-18	Acute appendicitis	
	M	27	Bangladeshi	19-Sep-18	Acute appendicitis	
	M	18	BAH	20-Sep-18	Early acute appendicitis	
	M	23	Indian	20-Sep-18	Acute appendicitis	
	F	32	BAH	21-Sep-18	Acute appendicitis	
	M	29	Bangladeshi	22-Sep-18	Acute appendicitis	
	M	20	BAH	22-Sep-18	Acute appendicitis	
	M	20	BAH	24-Sep-18	Reactive lymphoid hyperplasia	
	M	25	BAH	24-Sep-18	Acute appendicitis	
	F	18	BAH	25-Sep-18	Early acute appendicitis	
	M	51	BAH	25-Sep-18	Acute appendicitis	
	M	22	Indian	25-Sep-18	Acute appendicitis	
	M	8	BAH	25-Sep-18	Gangrenous appendix	Not specified
	F	23	BAH	27-Sep-18	Acute appendicitis	
	M	40	Bangladeshi	28-Sep-18	Acute appendicitis	
	F	28	Yemeni	29-Sep-18	Early acute appendicitis	
	M	34	Bangladeshi	30-Sep-18	Acute appendicitis	
	F	23	African	30-Sep-18	Acute appendicitis	
T2	F	29	European	4-Oct-18	Gangrenous appendix	Not specified
	F	28	BAH	5-Oct-18	Acute appendicitis	
	M	29	Bangladeshi	5-Oct-18	Acute appendicitis	
	M	24	Pakistani	5-Oct-18	Early acute appendicitis	
	M	31	BAH	5-Oct-18	Acute appendicitis	
	M	28	Bangladeshi	6-Oct-18	Acute appendicitis	
	M	26	BAH	6-Oct-18	Gangrenous appendix	Not specified
	M	33	Indian	7-Oct-18	Acute appendicitis	
	M	37	Pakistani	7-Oct-18	Normal	
	M	16	BAH	9-Oct-18	Acute appendicitis	
	M	31	Bangladeshi	11-Oct-18	Acute appendicitis	
	M	48	Indian	12-Oct-18	Early acute appendicitis	
	F	10	BAH	12-Oct-18	Reactive lymphoid hyperplasia	
	M	27	Bangladeshi	13-Oct-18	Acute appendicitis	
	M	33	BAH	14-Oct-18	Acute appendicitis	
	M	30	Bangladeshi	15-Oct-18	Acute appendicitis	
	M	17	BAH	15-Oct-18	Acute appendicitis	
	M	31	Pakistani	15-Oct-18	Early acute appendicitis	
	F	23	BAH	15-Oct-18	Acute appendicitis	
	F	13	BAH	15-Oct-18	Reactive lymphoid hyperplasia	
	M	31	BAH	16-Oct-18	Acute appendicitis	
	M	20	Pakistani	16-Oct-18	Gangrenous appendix	2
	M	40	Indian	17-Oct-18	Acute appendicitis	
	M	19	BAH	17-Oct-18	Acute appendicitis	
	M	30	Bangladeshi	17-Oct-18	Acute appendicitis	

M	6	Indian	17-Oct-18	Acute appendicitis	
M	35	Indian	18-Oct-18	Acute appendicitis	
M	26	BAH	18-Oct-18	Acute appendicitis	
M	30	Bangladeshi	19-Oct-18	Acute appendicitis	
M	32	GCC	20-Oct-18	Acute appendicitis	
M	6	GCC	20-Oct-18	Acute appendicitis	
M	47	BAH	21-Oct-18	Acute appendicitis	
F	44	Indian	21-Oct-18	Acute appendicitis	
F	18	BAH	22-Oct-18	Cicatrized	
M	35	BAH	23-Oct-18	Acute appendicitis	
M	28	Bangladeshi	24-Oct-18	Early acute appendicitis	
M	34	Bangladeshi	24-Oct-18	Acute appendicitis	
M	29	Indian	24-Oct-18	Acute appendicitis	
F	15	BAH	25-Oct-18	Early acute appendicitis	
M	14	BAH	25-Oct-18	Acute appendicitis	
M	37	Bangladeshi	25-Oct-18	Reactive lymphoid hyperplasia	
M	31	BAH	26-Oct-18	Early acute appendicitis	
M	35	Pakistani	26-Oct-18	Acute appendicitis	
M	27	Pakistani	26-Oct-18	Acute appendicitis	
M	27	Bangladeshi	26-Oct-18	Acute appendicitis	
M	21	Nepalese	26-Oct-18	Acute appendicitis	
M	6	BAH	26-Oct-18	Acute appendicitis	
F	21	BAH	27-Oct-18	Acute appendicitis	
M	45	Philipino	27-Oct-18	Acute appendicitis	
M	14	BAH	27-Oct-18	Acute appendicitis	
F	20	Indian	28-Oct-18	Acute appendicitis	
M	19	Indian	29-Oct-18	Acute appendicitis	
M	30	Indian	29-Oct-18	Acute appendicitis	
M	29	Indian	30-Oct-18	Acute appendicitis	
F	18	BAH	31-Oct-18	Acute appendicitis	
M	18	BAH	31-Oct-18	Cicatrized	
M	28	Bangladeshi	31-Oct-18	Early acute appendicitis	
M	49	Bangladeshi	1-Nov-18	Acute appendicitis	
M	21	BAH	2-Nov-18	Acute appendicitis	
M	58	BAH	2-Nov-18	Acute appendicitis	
M	21	Indian	3-Nov-18	Acute appendicitis	
M	41	Bangladeshi	3-Nov-18	Acute appendicitis	
F	36	Philipino	5-Nov-18	Acute appendicitis	
M	24	Pakistani	6-Nov-18	Acute appendicitis	
M	35	Bangladeshi	6-Nov-18	Acute appendicitis	
F	43	Philipino	8-Nov-18	Acute appendicitis	
M	30	Pakistani	8-Nov-18	Reactive lymphoid hyperplasia	
M	52	BAH	9-Nov-18	Acute appendicitis	
M	36	Bangladeshi	9-Nov-18	Acute appendicitis	
M	26	Indian	10-Nov-18	Acute appendicitis	
M	29	Bangladeshi	11-Nov-18	Acute appendicitis	
M	57	BAH	12-Nov-18	Gangrenous appendix	10
F	25	African	12-Nov-18	Acute appendicitis	
M	51	Bangladeshi	12-Nov-18	Acute appendicitis	
M	34	Bangladeshi	12-Nov-18	Acute appendicitis	
M	30	Indian	13-Nov-18	Acute appendicitis	
F	20	BAH	13-Nov-18	Acute appendicitis	

	M	27	BAH	14-Nov-18	Acute appendicitis	
	M	31	Bangladeshi	16-Nov-18	Acute appendicitis	
	M	31	Bangladeshi	18-Nov-18	Acute appendicitis	
	M	39	GCC	19-Nov-18	Early acute appendicitis	
	M	26	Indian	19-Nov-18	Acute appendicitis	
	M	27	BAH	21-Nov-18	Acute appendicitis	
	F	15	BAH	22-Nov-18	Acute appendicitis	
	M	25	Indian	22-Nov-18	Acute appendicitis	
	M	25	BAH	23-Nov-18	Acute appendicitis	
	M	33	Nepalese	23-Nov-18	Acute appendicitis	
	F	12	Indian	23-Nov-18	Reactive lymphoid hyperplasia	
	M	27	Indian	24-Nov-18	Acute appendicitis	
	M	17	BAH	24-Nov-18	Acute appendicitis	
	M	21	Pakistani	24-Nov-18	Early acute appendicitis	
	F	25	African	24-Nov-18	Reactive lymphoid hyperplasia	
	M	27	BAH	24-Nov-18	Gangrenous appendix	1
	M	29	BAH	24-Nov-18	Acute appendicitis	
	M	25	Indian	26-Nov-18	Acute appendicitis	
	F	32	BAH	28-Nov-18	Acute appendicitis	
	M	24	Indian	28-Nov-18	Acute appendicitis	
	M	36	Indian	28-Nov-18	Acute appendicitis	
	F	24	BAH	28-Nov-18	Acute appendicitis	
	F	65	BAH	29-Nov-18	SSA	
	M	19	BAH	29-Nov-18	Acute appendicitis	
	F	34	BAH	30-Nov-18	Acute appendicitis	
	M	33	Pakistani	30-Nov-18	Acute appendicitis	
W	M	38	Philipino	2-Dec-18	Gangrenous appendix	1
	M	30	Indian	2-Dec-18	Acute appendicitis	
	F	21	Indonesian	3-Dec-18	Acute appendicitis	
	M	27	Indian	4-Dec-18	Early acute appendicitis	
	M	31	Pakistani	4-Dec-18	Gangrenous appendix	2
	M	25	Pakistani	5-Dec-18	Acute appendicitis	
	M	13	Indian	5-Dec-18	Acute appendicitis	
	M	31	Bangladeshi	6-Dec-18	Gangrenous appendix	2
	M	22	Moroccan	7-Dec-18	Gangrenous appendix	1
	F	40	Moroccan	7-Dec-18	Acute appendicitis	
	M	25	BAH	7-Dec-18	Cicatrized	
	M	26	BAH	7-Dec-18	Early acute appendicitis	
	M	7	BAH	7-Dec-18	Gangrenous appendix	3
	F	42	BAH	8-Dec-18	Acute appendicitis	
	M	32	Philipino	9-Dec-18	Acute appendicitis	
	F	20	BAH	9-Dec-18	Acute appendicitis	
	F	11	BAH	9-Dec-18	Acute appendicitis	
	M	31	Bangladeshi	10-Dec-18	Acute appendicitis	
	F	33	BAH	10-Dec-18	Acute appendicitis	
	M	34	Bangladeshi	11-Dec-18	Acute appendicitis	
	M	38	Bangladeshi	11-Dec-18	Acute appendicitis	
	F	27	African	11-Dec-18	Early acute appendicitis	
	M	30	Pakistani	12-Dec-18	Acute appendicitis	
	M	35	BAH	12-Dec-18	Acute appendicitis	
	M	35	Bangladeshi	13-Dec-18	Acute appendicitis	
	F	27	African	14-Dec-18	Acute appendicitis	

	M	56	BAH	14-Dec-18	Acute appendicitis	
	M	35	Bangladeshi	15-Dec-18	Acute appendicitis	
	M	9	BAH	15-Dec-18	Acute appendicitis	
	F	24	BAH	17-Dec-18	Cicatrized	
	M	15	Indian	17-Dec-18	Early acute appendicitis	
	M	26	BAH	19-Dec-18	Acute appendicitis	
	M	66	BAH	20-Dec-18	Acute appendicitis	
	M	21	Indian	21-Dec-18	Acute appendicitis	
	M	22	BAH	21-Dec-18	Gangrenous appendix	1
	M	32	BAH	23-Dec-18	Acute appendicitis	
	M	14	BAH	23-Dec-18	Early acute appendicitis	
	M	30	Philipino	24-Dec-18	Acute appendicitis	
	F	22	Indian	24-Dec-18	Acute appendicitis	
	M	25	Pakistani	24-Dec-18	Acute appendicitis	
	M	24	Nepalese	25-Dec-18	Acute appendicitis	
	M	12	BAH	25-Dec-18	Acute appendicitis	
	M	38	Bangladeshi	27-Dec-18	Acute appendicitis	
	M	25	Indian	27-Dec-18	Acute appendicitis	
	M	33	Nepalese	27-Dec-18	Early acute appendicitis	
	M	32	Bangladeshi	27-Dec-18	Acute appendicitis	
	F	24	BAH	28-Dec-18	Reactive lymphoid hyperplasia	
	M	21	Indian	28-Dec-18	Acute appendicitis	
	M	29	Nepalese	28-Dec-18	Acute appendicitis	
	M	25	BAH	30-Dec-18	Acute appendicitis	
	M	18	BAH	30-Dec-18	Acute appendicitis	
